# Supplementary material for: State-switching and high-order spatiotemporal organization of dynamic functional connectivity are disrupted by Alzheimer’s disease
Source: Netw Neurosci. 2023 Dec 22;7(4):1420–51. doi: 10.1162/netn_a_00332 (PMC10727776; doi:10.1162/netn_a_00332)
Supplement: Supplementary file 1 [file netn-7-4-1420-s001.pdf]

Supplementary tables

Table S1. Differential impact of pathology on FC dimers and MC trimers and tetramers.

	Intra-zone			
	SNC	NC	aMCI	AD
FC	0.543±0.170	0.564±0.155	0.549±0.186	0.490±0.180
Trimers	0.359±0.139	0.348±0.126	0.333±0.146	0.318±0.144
Tetramers***	0.222±0.096	0.196±0.087	0.186±0.077	0.156±0.088
	Inter-zone			
FC**	0.101±0.114	0.083±0.135	0.054±0.126	0.021±0.088
Trimers**	0.039±0.078	0.019±0.083	0.013±0.072	-0.012±0.052
Tetramers***	0.183±0.134	0.187±0.117	0.138±0.137	0.139±0.120

*Average strengths of dimer, trimer and tetramer interactions, by clinical group and relation to anatomical zones. Values are means ± SD; * significantly inter-group variations with P < 0.05; ** with P < 0.01; *** with P < 0.001 (one-way ANOVA test).*

Table S2. Memory coefficients for dynamic links in the four groups

	Intra-zone			
	SNC	NC	aMCI	AD
5%	0.1561	0.1310	0.1168	0.1037
50%	0.1653	0.1383	0.1238	0.1098
95%	0.1746	0.1457	0.1307	0.1158
	Inter-zone			
5%	0.1407	0.1391	0.1404	0.0901
50%	0.1452	0.1428	0.1443	0.0928
95%	0.1498	0.1465	0.1481	0.0954

The memory coefficient, by clinical group and relation to anatomical zones. Values are means and the confidence intervals; Intra-zone: SNC >>> NC, aMCI >>> NC, AD >>> NC ; Inter-zone : aMCI >>> NC, AD >>> NC ; where, >>> means p-value smaller than 0.001.

Supplementary figures

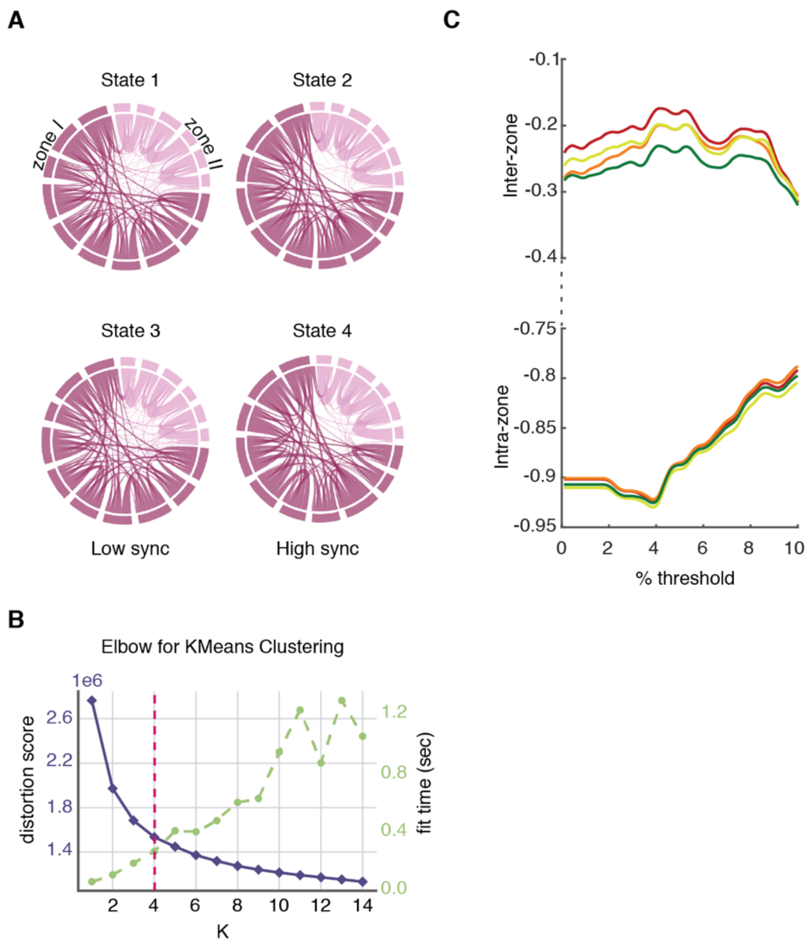

Fig. S1. Addition information on state-based dFC analyses. (A) Chord diagrams of $FC^{(\lambda)}$ states as an alternative illustration of Fig. 1A. Dark pink regions correspond to Zone I and light pink regions to Zone II. States 1 and 3 with low synchronization have stronger inter-zone connections than states 2 and 4 with high synchronization. **(B)** We used an elbow criterion based on the Silhouette score to guess the optimal number of clusters. The distortion (linked to the distance between cluster centroids) slows down its decrease with *k* while the time of clustering keeps growing, leadings to estimate a number of retained clusters around four (**C**). We show here the dependence of the average burstiness coefficient β for all groups on different choices of binarization thresholds θ. which were averaged over dFC dimers into

two intra- and inter-zone categories of links is shown (colored solid lines; green: SNC, yellow: NC, orange: aMCI, red: AD). The fact that the gap and the relative ranking between curves for the different groups remain consistent over different thresholds justifies the use of relative excess values for the analyses of Figure 3E.

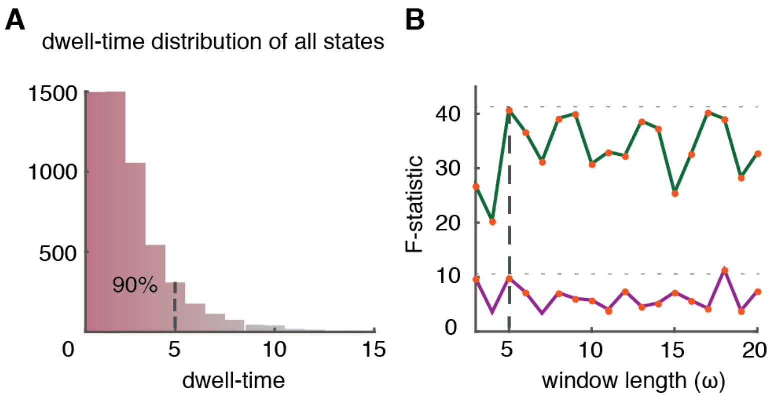

Fig. S2. Length of window in MC approach. (**A**) Distribution of the duration of mean dwell-times in a consistent state (from the state-based PBM method), pooled over subjects and states (see Fig 2C). We see that ~90% of epochs last less than 5 TRs. (**B**) We applied one-way ANOVA on average MC strengths to determine the existence of inter-group differences. Shown here is the value of the F-statistic for existence of inter-group differences, as a function of changing window size, from 3TRs to 20TRs. We performed the analysis separately for *intra-zone* (green line) and *inter-zone* (violet line) subsets of trimers. Using larger windows would not improve the statistical detection of inter-group differences. A short window of length ω = 5TRs is thus already sufficient to capture between-group differences, maintaining at the same time the capability to track the very fast dFC fluctuations revealed by Fig. S2A.

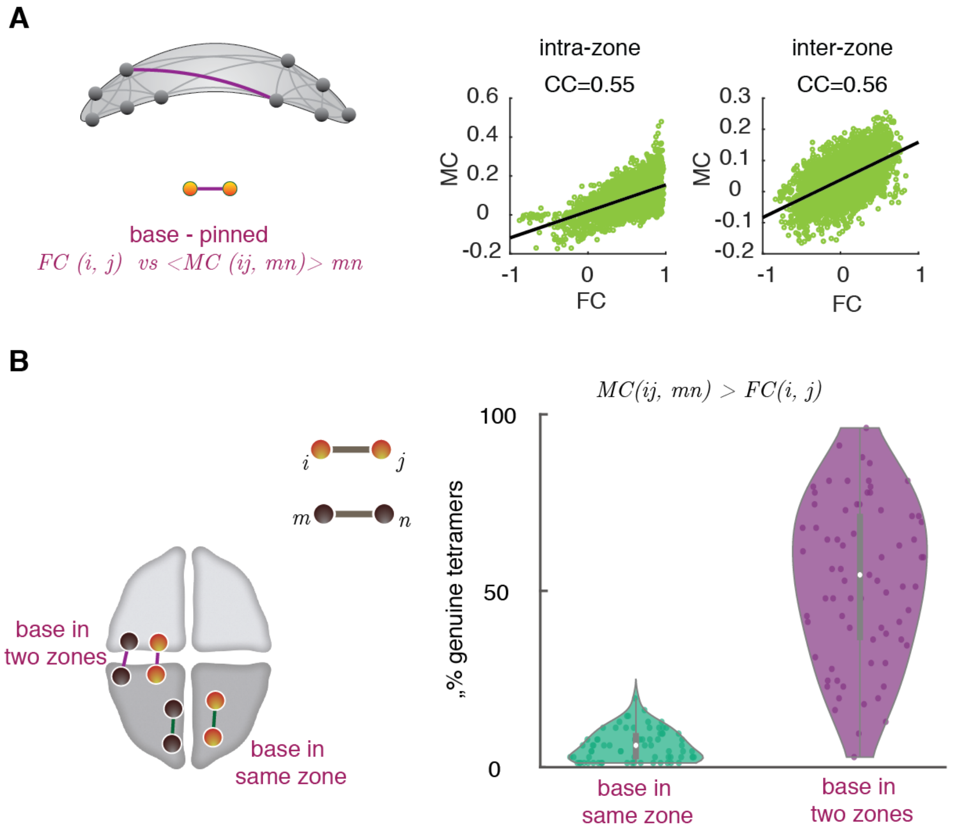

Fig. S3. State-free dFC: Inter-relations between dFC tetramers and FC dimers. (**A**) Similarly to the MC-FC comparison at the trimer level (see Fig. 5A), we compared dimer and tetramer strengths now for edges. The scatter plots show values of FC dimers paired with the corresponding base-pinned tetramer strength of that dimer (i.e. the overall meta-coupling of that dimer to other remote and non-incident dimers). Again, values are separated for intra- and inter-zone dimers and tetramers. Unlike for trimers, strong dimers are also the ones with the strongest tetramer strengths, as revealed by significant positive correlations. (**B**) Generalizing Fig. 5B for trimers, we also computed the fraction of genuine tetramers. The *base in same zone* subset of tetramers contained a low fraction of genuine tetramers, while this fraction raised for tetramers with an inter-zone base.

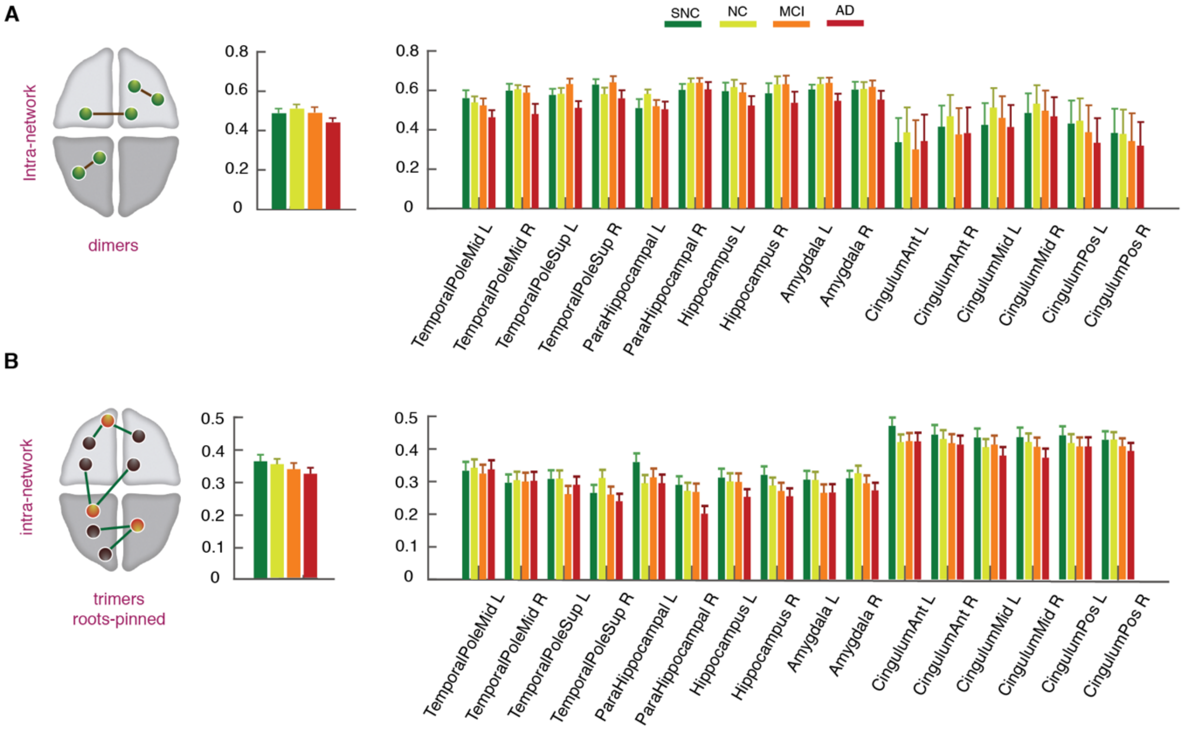

Fig. S4. State-free dFC: intra-zone FC dimers and dFC trimers strengths. (**A**) and (**B**) The FC dimers and dFC trimers for the intra-zone subset did not show any significant reduction of strength from SNC-to-AD group, despite moderately decreasing average values, both globally (left) and locally at the single region level (right).
